# Is cushing's syndrome remission associated with diabetes regression? Analysis of a retrospective cohort of 108 patients with cushing's disease

**DOI:** 10.1186/1758-5996-7-S1-A106

**Published:** 2015-11-11

**Authors:** Thizá Massaia Londero, Ana Marina da Silva Moreira, Sheila Piccoli Garcia, Fabíola Costenaro, Iuri Martin Goemann, Gustavo da Fonseca Cipriani, Camila Viecceli, Ticiana da Costa Rodrigues, Mauro Antônio Czepielewski

**Affiliations:** 1 Hospital de Clinicas de Porto Alegre, Porto Alegre, Brazil

## Background

Diabetes mellitus (DM) is a common comorbidity of Cushing's syndrome (CS) and plays an important role in morbidity and death of patients with uncontrolled hypercortisolism. Some authors define DM in CS as a ‘specific type of diabetes secondary to endocrinopathy’, although others judge it as a classical form of type 2 DM. Glucocorticoid (GC) excess causes pancreatic beta cell dysfunction and insulin resistance, which correlates with hypercortisolism level. If Cushing's disease (CD) remission implicates on DM resolution remains unclear.

## Objectives

To asses DM prevalence in CD patients and DM resolution rate after one year remission of CD.

## Materials and methods

Retrospective cohort of 108 patients diagnosed with CD between 1987 and 2014 at a tertiary endocrinology service. Patients underwent clinical and metabolic evaluation at diagnosis and after CD treatment. CD remission criteria after transsphenoidal surgery were: cortisol < 3 mcg/dl on the 1mg-overnight test, normal urinary free cortisol and/or adrenal insufficiency with GC dependence for 6 months. DM resolution criteria were HbA1c <6.5% and fasting glucose < 126mg/dL without antidiabetic drugs.

## Results

**Figure 1:**
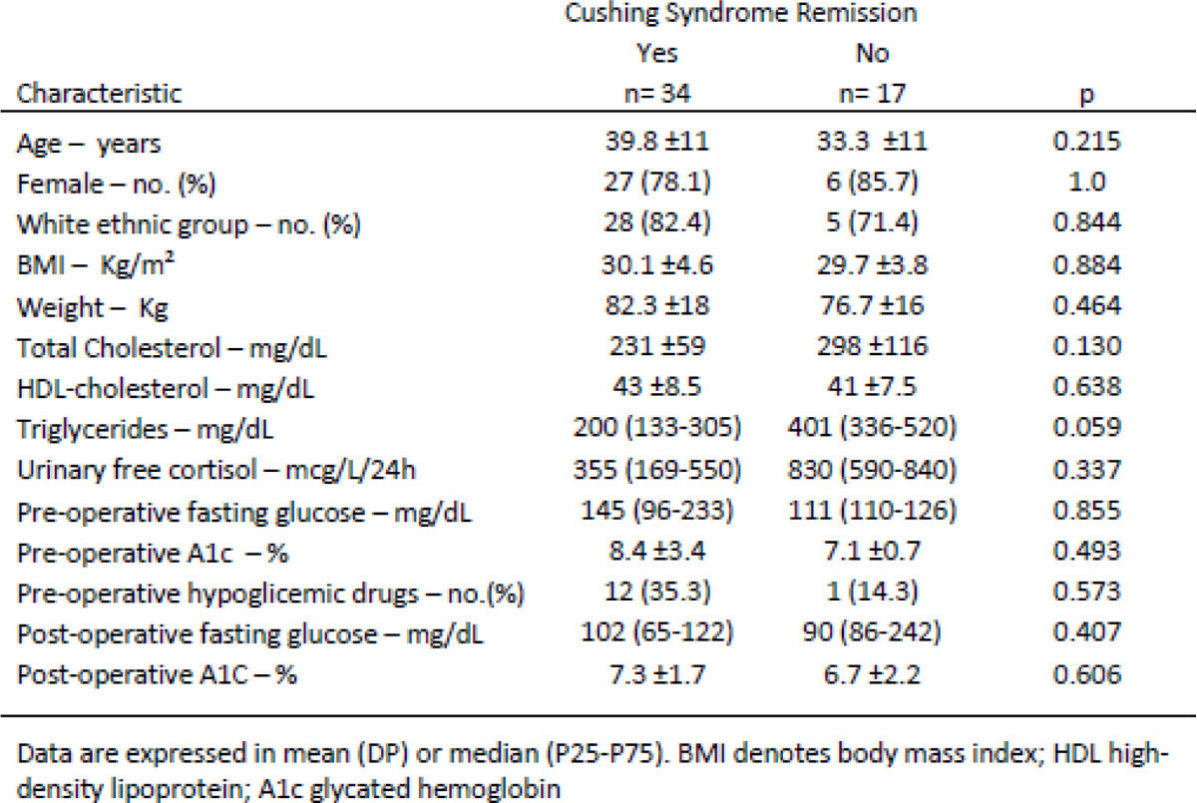
**Clinical and Biochemical Features of the Patients with Cushing's disease and DM** Data are expressed in mean (DP) or median (P25-P75). BMI denotes body mass index; HDL high-density lipoprotein; A1c glycated hemoglobin

Patients clinical and biochemical features are presented in Figure 1. Of the 108 CD patients, 38% had DM diagnosis and 32% were treated with hypoglycemic agents and/or insulin. CD remission was achieved in 73% of the diabetic patients and 66% were also considered cured of DM after 1 year (p=0.378). There was no statistically significant association between age, gender, body mass index, lipid profile, 24h-UFC at diagnosis and DM regression.

## Discussion

In this representative CD sample, prevalence of DM was consistent with literature, ranging from 30-50%. Only a few reports analyze DM persistence after CD resolution and possible cure predictors. No relationship was found between DM and CD short term remission. Dysglycemia may persist up to 5 yrs. after CD treatment, showing a metabolic imprinting of chronic GC excess. DM resolution also depends on the weight loss, physical activity, body composition and prolonged use of GC following surgery.

## Conclusion

DM affects up to 40% of patients with CD and its short term remission does not seem to predict the resolution of diabetes, perhaps because metabolic effects persists even after correction of hypercortisolism and factors associated with DM cure are heterogeneous.

